# Why and How Savitzky–Golay Filters Should Be Replaced

**DOI:** 10.1021/acsmeasuresciau.1c00054

**Published:** 2022-02-18

**Authors:** Michael Schmid, David Rath, Ulrike Diebold

**Affiliations:** Institute of Applied Physics, TU Wien, 1040 Vienna, Austria

**Keywords:** data smoothing, Savitzky−Golay filter, spectra processing, differentiation, noise suppression

## Abstract

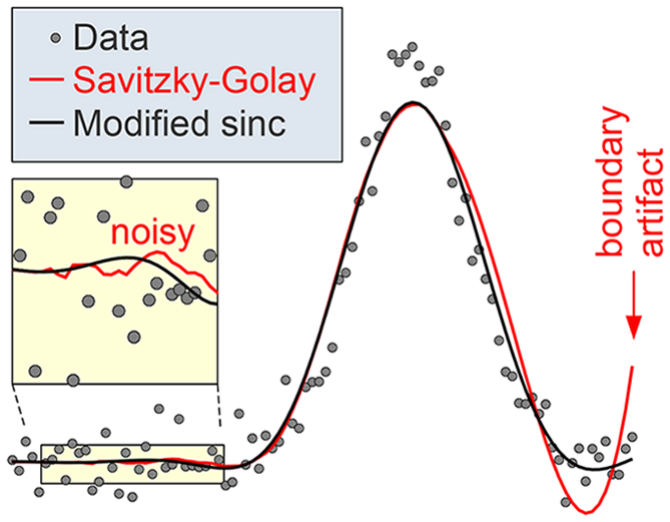

Savitzky–Golay (SG) filtering, based on local least-squares fitting of the data by polynomials, is a popular method for smoothing data and calculations of derivatives of noisy data. At frequencies above the cutoff, SG filters have poor noise suppression; this unnecessarily reduces the signal-to-noise ratio, especially when calculating derivatives of the data. In addition, SG filtering near the boundaries of the data range is prone to artifacts, which are especially strong when using SG filters for calculating derivatives of the data. We show how these disadvantages can be avoided while keeping the advantageous properties of SG filters. We present two classes of finite impulse response (FIR) filters with substantially improved frequency response: (i) SG filters with fitting weights in the shape of a window function and (ii) convolution kernels based on the sinc function with a Gaussian-like window function and additional corrections for improving the frequency response in the passband (modified sinc kernel). Compared with standard SG filters, the only price to pay for the improvement is a moderate increase in the kernel size. Smoothing at the boundaries of the data can be improved with a non-FIR method, the Whittaker–Henderson smoother, or by linear extrapolation of the data, followed by convolution with a modified sinc kernel, and we show that the latter is preferable in most cases. We provide computer programs and equations for the smoothing parameters of these smoothers when used as plug-in replacements for SG filters and describe how to choose smoothing parameters to preserve peak heights in spectra.

## 1 Introduction

Since their introduction more than half a century ago,^1^ Savitzky–Golay (SG) filters have been popular in many fields of data processing; ranging from spectra in analytical chemistry^2−4^ via geosciences^5^ to medicine.^6,7^ SG filters are usually applied to equidistant data points and are based on fitting a polynomial of given degree *n* to the data in a (usually symmetric) neighborhood *k* – *m*...*k* + *m* of each data point *k* (this range contains 2*m* + 1 data points). For smoothing the data, each data point is replaced by the value of the fit polynomial at this point *k*;^8^ alternatively, a derivative of the polynomial can be used to obtain a smoothed derivative. As this process is a linear filter and takes a limited number of points as the input, SG smoothing is a finite impulse response (FIR) filter. Therefore, it can be implemented as a convolution with a suitable kernel.^1^ The SG kernels can be calculated numerically^9^ or from analytical formulas.^10,11^ The same applies to SG filters for the calculation of derivatives. In the frequency domain, SG smoothing filters have a flat passband and a rather steep cutoff, with the steepness increasing with the degree of the fit polynomial.^8^ For simplicity, we will simply write “degree of the filter” for the degree of the fit polynomial used in the following. (We use the word “degree”, not “order”, as order often refers to the length of the kernel of an FIR filter.) The flat passband and steep cutoff leads to an advantageous property of SG filters when smoothing spectra: SG smoothing filters preserve peaks and their heights better than many other filters with a similar cutoff frequency (see section 3.1).

**Figure 1:**
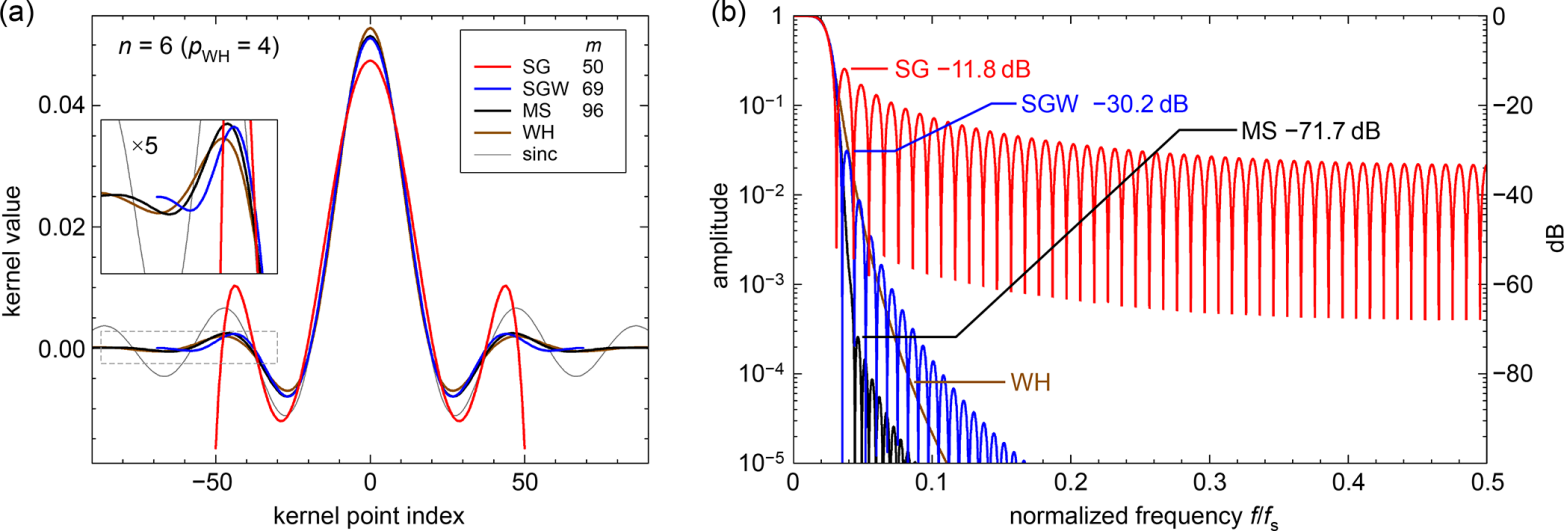
Kernels and frequency response of the different filter types. Comparison of an *n* = 6 (6th degree) SG filter and other smoothing filters with similar frequency response in the passband: SG with Hann-square fitting weights (SGW), modified sinc kernel (MS), and Whittaker–Henderson (WH). The half-widths *m* of the kernels (λ value for WH) were chosen for a similar cutoff *f*_–3dB_ in the frequency domain. (a) Convolution kernels of these filters (response to a unit impulse for WH); a sinc function without windowing is shown for comparison. The inset shows a magnified region. (b) Frequency response of the filters; *f*_s_ is the sampling frequency. Except for WH, the amplitude of the first sidelobe is indicated for each filter. To avoid cluttering, the minima between the SG sidelobes have been truncated; there, the frequency response reaches zero. For other degrees *n*, see Figure 2 and Figures S1 and S2 in the Supporting Information.

SG filters have an unsatisfactory frequency response in the stopband, though: high-frequency noise is not efficiently suppressed. The attenuation above the cutoff frequency, at the first sidelobe of the frequency response, is only −11 to −13 dB (amplitude attenuation to about 1/4);^8^ see Figure 1b. The higher sidelobes have less amplitude, but the amplitude decay is only about 1/*f*. The poor stopband suppression is almost independent of the kernel half-width *m* (*m* determines the cutoff frequency); see Figure S3. We will show that the insufficient attenuation in the stopband poses a problem, especially if the derivative of the data is of interest. This property of SG filters makes their use for determining higher derivatives impractical. The reason for this unsatisfactory suppression of high frequencies becomes intuitively clear when looking at the convolution kernel of an SG filter, shown in Figure 1a, for a 6th degree SG filter with large *m* (where the kernel, though consisting of discrete points, can be considered an almost continuous function).

The SG kernel (red curve) is discontinuous at −*m* and *m*; there is a sudden jump from a rather large value to zero. In addition, the kernel shows undesirably rapid oscillations near the end points. This becomes obvious when comparing with a sinc function, that is, a kernel with an ideally sharp cutoff from 1 to 0 at the same cutoff frequency (thin gray line): the last positive half-wave of the SG kernel in Figure 1a has a width that is only about half of what it should be (the half-wave of the sinc kernel), and the negative excursion toward the discontinuity is extremely steep. Therefore, it is obvious that the SG kernel contains strong Fourier components at frequencies above the cutoff frequency (the cutoff corresponds to the wavelength of the wiggles of the sinc function). Convolution with a kernel will multiply the frequency spectrum of the data with that of the kernel; therefore, smoothing with a SG filter does not suppress the high-frequency Fourier components of the data as much as one would desire.

When reading the literature about SG filters, one could get the impression that the unsatisfactory suppression of frequencies above the cutoff is the price to pay for the flat frequency response in the passband, and that the room for improvements is limited.^8,12^ Most recent developments regarding SG filters focus on adaptive SG filtering and adjusting the filter parameters to best fit the signal and noise of a given data set,^5,6,13^ not on improving the filter. In this paper, we show that a flat passband can still be achieved while the stopband is strongly attenuated. We present two new approaches and one known solution for this problem and discuss the respective merits and disadvantages: (i) We show that choosing suitable weights for the fit can substantially improve the stopband attenuation of SG filters by removing the discontinuity at the ends of the kernel. (ii) A convolution kernel based on a sinc function with a Gaussian-like window has excellent suppression in the stopband. (iii) SG smoothing can also be replaced by the Whittaker–Henderson smoothing algorithm.^14−16^ We analyze the near-boundary behavior of these methods, their noise suppression, and their suitability for calculating derivatives. Filter kernels (unit impulse responses) and the corresponding frequency responses of these types are also shown in Figure 1 (“SGW” for Savitzky–Golay with weights, “MS” for modified sinc kernel, and “WH” for Whittaker–Henderson).

## 2 Filters That Can Replace Savitzky–Golay

### 2.1 Savitzky–Golay Filters with Fitting Weights (SGW)

A simple way of improving the SG filters is applying weights *w* when fitting to reduce the influence of the data points at the periphery of the *k* – *m*...*k* + *m* interval on the fit. Astonishingly, this is rarely mentioned in the literature,^17,18^ and to our knowledge, there has been no in-depth study of this possibility. Essentially all window functions^19^ used for kernel construction and Fourier transforms are suitable as weights, as long as they do not contain negative values. As a rule of thumb, window functions with smoothness of high derivatives and good sidelobe suppression in the Fourier domain also provide low sidelobes when used as SGW weights. For *n* = 6 and sufficiently large *m*, we obtain first sidelobe amplitudes of −22.0, −21.1, −30.2, and −39.2 dB for the Gaussian-based window discussed in section 2.2 with α = 2, the Hann, Hann-square (also known as cos^4^ window) and Hann-cube (cos^6^) windows, respectively (see ref (19) for the cos-based window functions). In the following, we will use the Hann-square (cos^4^) function for the weights. Figure 1 shows that the SGW kernel (blue) is continuous at the ends (as are its lowest three derivatives), and that suppression of the stopband is substantially improved. For achieving a cutoff frequency comparable to the SG filter, the kernel size required for the SGW filter is about 30–70% larger (depending on *n*), which is a moderate price to pay for the improvement. In the passband, the SGW and SG filters have almost identical frequency response; for degree *n*, both have an initial 1 – *cf*^*n*+2^ transmission^8^ where *c* is a constant.

In contrast to traditional SG filters (and the MS kernels described below), there are no simple algebraic equations for the kernel functions of the SGW filters. Nevertheless, it is easy to construct these kernels by starting with (modified) Gram–Schmidt orthogonalization to create a set of *n* + 1 orthonormal polynomials *p*_*j*_(*i*) of degree *j* = 0, 1,...*n*. These polynomials fulfill
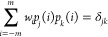
1For a constant weight function, orthonormalization would result in Chebyshev’s “discrete orthogonal polynomials”, which can be used to construct the traditional SG kernels.^11^ The kernel coefficients *a*_*i*_ can be obtained via
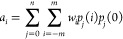
2Except at the boundaries (see below), where the sum over *i* does not run over the full −*m*...*m* range, it is sufficient to use only polynomials of even degree *j*; the contributions of the odd polynomials vanish if the window function has even symmetry.

### 2.2 Convolution Kernels Based on the Sinc Function (MS)

The sinc function sin *x*/*x* has a rectangular Fourier transform, that is, a perfectly flat passband, and a sharp transition to zero response in the stopband. Due to its infinite extension and slow 1/*x* decay, it is not directly usable as a convolution kernel; for application as a kernel, it has to be multiplied with a suitable window function *w*.^19^ Gaussian windows have the advantage of having the minimum time-bandwidth product (for the mean-square time and bandwidth values, see ref (19)), and they also show fast decay. Multiplication of the kernel corresponds to convolution in the Fourier domain. When multiplying a sinc function with a Gaussian window, we get an amplitude decay corresponding to a complementary error function (erfc) instead of a sharp frequency cutoff. A Gaussian window does not have a finite width, however. Truncating it to get a finite kernel (FIR filter) would cause a discontinuity, which increases the high-frequency Fourier components. Modifications of the Gaussian window for minimizing the kernel size are known from ref (20). For our application, we put emphasis on optimizing the stopband suppression; we take a different (though related) approach. We modify the Gaussian window by adding two out-of-window Gaussians and an offset in such a way that, at the end points of the window, the sum has not only zero amplitude but also an almost-zero derivative (supporting Figure S4). We also choose the sinc function such that the end points of the window coincide with a zero of the sinc function. The kernel and its first derivative are exactly zero at the end points, and its second derivative is extremely close to zero.^21^ This results in the following kernel function *a*:
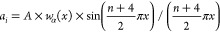
3with the window function
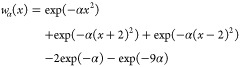
4where
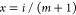
5is zero in the center of the kernel and *x* = ±1 at the first point outside the kernel, that is, where both the sinc and the window function reach a value of zero (*i* runs from −*m* to *m*). The index *n* must be even and determines how many wiggles of the sinc function the kernel contains. For *n* = 2, we have one minimum and one maximum at each side; for *n* = 4, also the next minimum is included, etc. The MS kernels have *n* + 2 inner zeros (i.e., not counting the zero at each end). As the MS kernels are wider than the comparable SG and SGW kernels (*n* inner zeros), the MS kernels can achieve better stopband suppression (see below). For the Gaussians in eq 4, α determines the width of the Gaussians and thus the steepness of the cutoff in the frequency domain (the erfc function mentioned above); we chose α = 4 for a steepness similar to that of the SG or SGW filters with the same *n*. Finally, the normalization factor *A* must be chosen such that the sum over the kernel coefficients fulfills
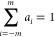
6

For *n* ≥ 6, the kernels described by eq 3 are not perfect in the passband, however. We find up to ≈0.13% (0.01 dB) overshoot for *n* ≥ 6. This may be tolerable in most cases, but it is not as perfect as the flat passband of the SG filters. The reason for the imperfect passband lies in an imbalance of the side maxima and minima of the sinc function, caused by the window and truncation. We can improve the passband response by adding small correction terms, replacing eq 3 with
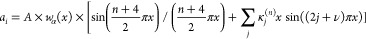
7where ν = 1 for odd *n*/2, i.e., *n* = 6 or 10 and ν = 2 otherwise (*n* = 8). Like the sinc function, the correction terms in the second line of eq 7 are zero at the edge of the kernel (*x* = ± 1), so they do not compromise the continuity of the kernel and its derivatives at the edge; this is required for good attenuation in the stopband. We have determined suitable values of the correction coefficients κ_*j*_^(*n*)^ for *n* = 6, 8, and 10, and *m* ≥ *n*/2 + 2 by minimizing a weighted sum of the square deviations from an ideal flatband response. We found that these optimum κ_*j*_^(*n*)^ values can be fitted by
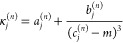
8The coefficients *a*_*j*_^(*n*)^, *b*_*j*_^(*n*)^, and *c*_*j*_^(*n*)^ found by our minimization procedure are listed in Table 1. With these corrections, we obtain a flat frequency response in the passband with negligible overshoot (<3.5 × 10^–6^, corresponding to <0.00003 dB).

**Table 1:** Coefficients in Equation 8^a^

*n*	6	8	8	10	10
*j*	0	0	1	0	1
*a*_*j*_^(*n*)^	0.00172	0.00440	0.00615	0.00118	0.00367
*b*_*j*_^(*n*)^	0.02437	0.08821	0.02472	0.04219	0.12780
*c*_*j*_^(*n*)^	1.64375	2.35938	3.63594	2.74688	2.77031

a Which yield the correction terms for eq 7, to ensure a flat passband of the modified sinc kernels (for *n* ≤ 4, no such correction is needed).

**Figure 2:**
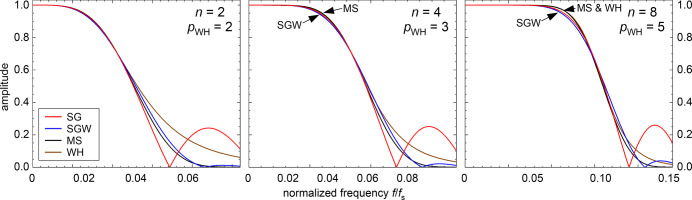
Frequency response of the filters with different degree *n* = 2,4, and 8. For the traditional SG filters, the kernel half-width was chosen as *m* = 50. The half-width *m* of the other kernels and λ for the WH filter were chosen to obtain the same cutoff frequency *f*_–3dB_ as the respective SG filter.

We name the filters based on these kernels of eq 7 “modified sinc kernels” (MS); their frequency response with *n* = 2, 4, and 8 is shown in Figure 2; logarithmic plots are in Figure 1b and supporting Figure S2. The stopband rejection of the MS kernels is excellent, with the first sidelobe below 3 × 10^–4^ (−70 dB). The performance of the MS kernels is also much better than that of the Lanczos kernels, which are popular for resampling in image processing.^22^ The Lanczos kernels have the first sidelobe slightly above −40 dB and about 1% waviness in the passband. The MS kernels also have lower sidelobes than the SGW kernels presented in section 2.1, at the cost of somewhat larger kernel size (about twice the kernel size of SG filters with the same cutoff frequency).

We also constructed modified sinc kernels with smaller sizes. Their stopband suppression is still substantially improved compared to the non-MS filters and good enough for almost all applications. These kernels, named “MS1”, are described in the Supporting Information. Finally, we should mention that the MS and MS1 kernels presented here use integer steps for the parameter *m*, which controls the smoothness. This is usually sufficient. (Due to the larger kernel size, MS is more fine-grained than for traditional SG filters.) If desired, it is possible to construct the same type of filters with a noninteger value corresponding to the zero at the end of the kernel, i.e., the *x* = ±1 points of eqs 4 and 7.

### 2.3 Whittaker–Henderson (WH) Smoothing

Whittaker–Henderson smoothing minimizes the functional
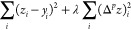
9where *y* are the data, *z* is the smoothed function, and Δ^*p*^*z* is the *p*th derivative of *z*, which is evaluated numerically.^14−16^ The sums over *i* run over all data points. (For the *p*th numeric derivative, the number of points is reduced by *p*.) Taking the *p*th derivative as an indication of smoothness, a penalty is imposed on nonsmooth functions, with higher values of λ increasing the penalty and therefore leading to smoother output.^23^ This method has been introduced several times, and it was generalized as smoothing splines since the 1960s^24^ and more recently popularized as “a perfect smoother”.^15^ WH smoothing is not an FIR filter; solving eq 9 for the smoothed function *z* leads to a matrix equation with a band-diagonal matrix that can be solved in *O*(*N*) time for *N* data points. For high degrees *p* and strong smoothing (very high values of λ), corresponding to large kernels for FIR filters, the WH smoother as implemented in ref (15) and the Java program in the Supporting Information suffers from numeric noise, however.^25^ For most practical applications, these high λ values are not required.

Far from the boundaries of the data set, WH smoothing behaves similar to an FIR filter (with a very large kernel),^26^ with the response to the unit impulse corresponding to the kernel (Figure 1). In this region, the frequency response of WH smoothing becomes
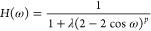
10with
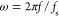
11where *f*_s_ is the sampling frequency.^27^ If a given cutoff frequency *f*_–3dB_ is desired, where the response decreases to , the smoothing parameter λ can be obtained from eq 10:
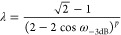
12

It follows from eq 10 that WH smoothing leads to a 1 – *cf*^2*p*^ response in the *f* → 0 limit. Comparison with the 1 – *cf*^*n*+2^ response of the SG and SGW filters shows that the low-frequency response has the same functional form if
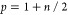
13Figures 1b and 2 show that WH smoothing with this choice of *p* yields an overall frequency response similar to that of the other filters of the corresponding degree *n* (also beyond the low-frequency limit). For *n* = 2 and 4 (*p* = 2 and 3), the WH attenuation in the stopband is somewhat weaker than that for the SGW and MS filters (Figure S2) . For *n* = 6 and 8 (and the corresponding *p* = 4, 5), the stopband attenuation of WH smoothing is still weaker than that for the MS convolution filter. (The SGW filter has a weaker stopband attenuation than MS.) Due to the very fast decay of the response with frequency, the signal in the stopband will be dominated by the frequencies close to the cutoff for both the WH and MS smoothing methods. Thus, the overall stopband rejection of WH smoothing is somewhat lower than that of the corresponding MS filters.

### 2.4 Replacing SG Filters

We provide readers with plug-in replacements for traditional Savitzky–Golay filters. For ready use, filter parameters need to give frequency characteristics similar to those of SG filtering (except for the poor stopband rejection of SG, of course, which our filters remedy). As discussed above, the frequency response of the SGW and MS filters is similar to that of SG if the degree *n* is the same. Thus, one can simply use the *n* value of the SG filter that should be replaced. For WH smoothing, the *p* parameter must be chosen according to eq 13. What remains is obtaining equal bandwidth *f*_–3dB_. An approximate equation for 2*f*_–3dB_/*f*_s_ of the SG filters for *m* ≥ 25 was given in ref (8). (Note that in that work, the frequency is defined with respect to the Nyquist frequency *f*_s_/2, not with respect to *f*_s_.) By least-squares fitting, we have obtained the following equation, which has sufficient accuracy for all *m* values:
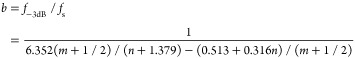
14For WH smoothing, the appropriate λ value is then given by eq 12. For the SGW and MS filters, least-squares fitting gives us the appropriate *m* values
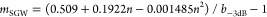
15and
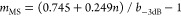
16These values should be rounded to the nearest integer to obtain the kernel half-width *m* that can be used for these filters. The filter parameters for Figures 1 and 2 have been obtained with these equations.

### 2.5 Filters Obtained by Sharpening

We should also mention that the alternatives to SG filtering discussed so far (SGW, MS, and WH) are not the only ones. Another possibility of obtaining a flat response in the passband and good suppression in the stopband is “sharpening” of a filter with a less sharp cutoff by linear combinations of single and multiple applications of the filter.^28,29^ This method works well if the filter it is based on has a reasonable stopband attenuation, better than that of traditional SG filters. Sharpening can be also based on convolution with a standard window function^19^ like the Hann or Hann-square window and obtain a frequency response comparable to that of the SG alternatives discussed here. By increasing the passband flatness, sharpening of a low-degree filter (e.g., SGW or MS with *n* = 2) can yield filter characteristics corresponding to a higher degree. We do not discuss this method in detail, as we could not find any advantage of sharpening over the filters discussed in the sections above. Neither would sharpening provide a solution for the near-boundary values discussed next.

### 2.6 The Problem of the Boundaries

Convolution with a kernel is only defined for the interior of the data series, not within a neighborhood of the kernel half-width *m* from the left and right boundaries. Especially for smoothing spectra with a finite length (given by the instrument), it is often desirable (or even required) to have smoothed data up to the boundaries of the input data. Conceptually, the SG filter seems well-suited for filtering the data near the boundaries: One can simply use the polynomial fit over the 2*m* + 1 neighborhood closest to the boundary for calculating the near-boundary values.^30^ This method is also used in the Matlab and GNU Octave *sgolayfilt* function.

**Figure 3:**
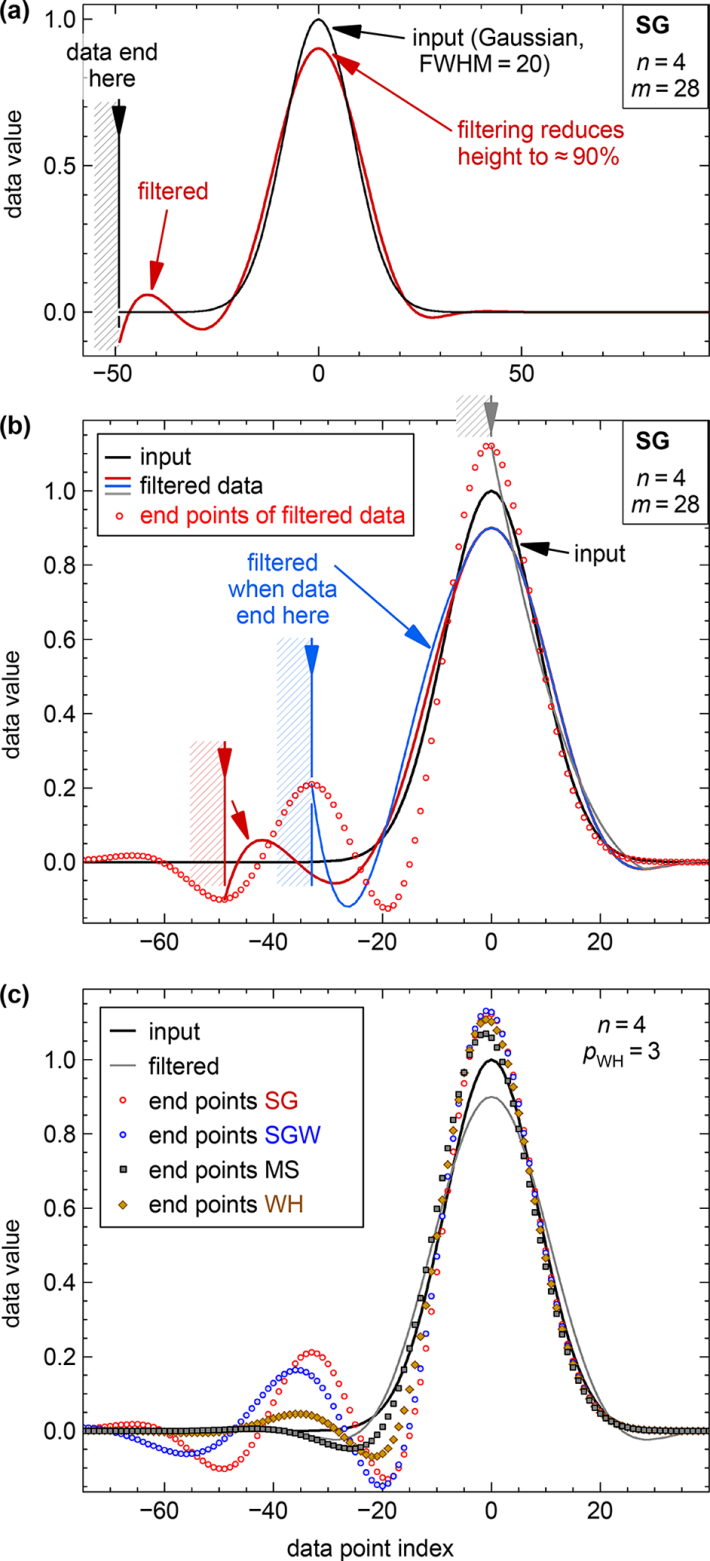
Filtering of a Gaussian peak near a boundary of the data. (a) Results of filtering a Gaussian peak by a traditional Savitzky–Golay (SG) filter (*n* = 4, *m* = 28), when the leftmost point of the input data is at −49. The SG-filtered data show strong ringing near the boundary. (b) Same as (a), with the data boundaries at three selected positions, marked by shaded areas with the same color as the respective curves [red, blue and gray; the red curve is the same as in (a)]. The red circles are the end points of the filtered data for all boundary positions of the input data. (c) End points of the filtered data for all boundary positions of the input data as in (b) but also including different filtering methods: Savitzky–Golay with Hann-square weights (SGW), the modified sinc kernel with linear extrapolation of the data (MS), and Whittaker–Henderson smoothing (WH). All filter parameters have been chosen such that the Gaussian would be smoothed to 90% of its peak height when not close to the boundary [gray curve in (c); this curve only weakly depends on the smoothing method; here, it is shown for MS convolution].

Compared to points in the interior of the data series, information is missing for calculating the smoothed values near or at the boundaries. On the one hand, this leads to a weaker suppression of noise than in the interior. On the other hand, artifacts may appear near the boundaries. In the following, we present test cases: as the input, we assume a Gaussian peak at or near the boundary. The smoothing parameters are chosen such that the Gaussian peak, when placed in the interior of the data, would be attenuated to 90% of its original height by smoothing. (We call this “90% peak height fidelity”. See section 3.1 for choosing the filter parameters to accomplish this.) This can be considered strong smoothing, which may be required for very noisy data, but not oversmoothing. An example of such a smoothing operation with a traditional SG filter is shown in Figure 3a. The input is a Gaussian with a full width at half-maximum (fwhm) of 20 (black curve), with the input data having 49 data points left of the peak. (At the right side, the data extend far enough to avoid any problems.) The SG-filtered curve shows a slight undershoot at the right side and an attenuation of the peak height, which would be the same for an equivalent Gaussian in the interior of the data. At the left side, in the region where the kernel reaches the boundary (since the boundary is at −50 and *m* = 28, this is for points below −22), the filtered curve does not follow the input and shows strong ringing artifacts.

Figure 3b shows the same for three different positions of the data boundary (red, blue, and gray). For the blue curve, where the input data reach −33 (i.e., 33 data points left of the peak and no input data in the blue shaded region), the behavior close to the boundary is undesirable and must be considered an artifact. The same procedure was applied for all possible positions of the left boundary of the data; the filtered curve was calculated for each of these cases. The end points of all these curves (including the red, blue, and gray ones) are plotted as red circles. Whereas the red and blue curves were chosen to show the most extreme cases of near-boundary artifacts, most other positions of the boundary also lead to unexpected, substantial artifacts unless the distance between the boundary and the center of the Gaussian is larger than about 60 points (this is more than the full width of the kernel, 2*m* + 1 = 57 in the present case).

As mentioned above, the treatment of the near-boundary data for the traditional SG filters is conceptually simple (polynomial fit to the 2*m* + 1 data values next to the boundary). For the SGW filters, the situation is not so straightforward. Simply shifting the weight function *w* would lead to weak smoothing at the boundary because *w* gets partially moved out of the range where data are available. Then the number of data points contributing to the end point would be almost half of what it is for interior points. Therefore, in analogy to the traditional SG, we choose to stretch the SGW weight function by a scale factor *s*, such that its sum over the range of valid data remains the same. Details of this procedure are described in the Supporting Information. With this choice of the weight function near the boundaries, the near-boundary artifacts of the SGW-smoothed data are lower than that with traditional SG smoothing; nevertheless, strong artifacts appear for some boundary positions. This can be seen in Figure 3c, which plots the end points of the filtered data for all positions of the left boundary for different smoothing methods. Figure 3 is for filters of degree *n* = 4, but similar artifacts also arise for other degrees (supporting Figure S5). For higher degrees *n*, the smoothed data better follow the input when the data boundary is inside the Gaussian peak. However, due to the larger kernel size, the artifacts reach boundary positions even further from the peak than for low *n*.

The WH smoothing method does not need any special treatment of near-boundary points; eq 9 is defined up to the boundaries of the data. (The numeric differentiation reduces the number of summands in the penalty term to *N* – *p* for *N* data points, but the filtered output *y* remains well-defined at all points.) Figures 3c and S5 also show the end points for smoothing with the WH method. (As for all filters, the end points show the near-boundary artifacts most clearly.) The artifacts are substantially reduced compared to the SG and SGW methods.

For convolution with the MS kernel up to the end, we have to extend the input data. One could mirror the input data at the boundary, but this would enforce the first derivative of the smoothed curve to vanish at the mirror position and create artifacts if the boundary is in a region where the slope of the data is nonzero. We have found that linear extrapolation of the data is a suitable method (extrapolation with a second-order polynomial is much worse). For the least-squares line fit, we use weights that decrease with increasing distance *i* from the boundary:
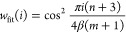
17if the argument of the cosine function is less than π/2; *w*_fit_ = 0 for higher arguments. This is one side of a Hann window function; for β = 1, it reaches zero close to the position of the first zero of the sinc function in the convolution kernel.

Of course, similar to filtering of interior points, also at the boundaries, there is a trade-off between noise suppression and fidelity in following the input data; the β parameter allows us to put more emphasis on one or the other for near-boundary points. Larger values of β increase the data range used for the linear regression and thus better suppress the noise, but the extrapolated curve will not necessarily reflect the trend of the data points close to the boundary. Therefore, also the smoothed curve will not nicely follow the shape of the input. We choose β such that the noise suppression at the boundaries is comparable to or better than that of the corresponding SGW or WH filters:
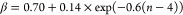
18The result is a good compromise: At the lowest degree, *n* = 2, the WH and MS filters show almost the same noise near the boundaries and have comparably low artifacts. For the higher degrees, the artifacts in the near-boundary region of the MS-filtered data are much less than for the other filters (see Figure 3c and Figure S5). Nevertheless, the noise suppression near the boundaries is better than that of the SGW or WH filters (see section 3.1). This extrapolation method can also be applied to other FIR filters like the SGW. We focus on the MS kernels due to their better stopband rejection.

The problem of artifacts at the boundaries is not limited to Gaussian peaks. As shown in the Supporting Information, the same occurs for smoothing Lorentzian peaks when the smoothing parameters are chosen such that the peak height gets attenuated by smoothing. All qualitative observations discussed above also apply for Lorentzian peaks. One should expect artifacts at the boundaries in all cases where the smoothed curve would not perfectly follow the input for interior points, or, in other words, when the input signal contains frequency components where the filter’s frequency response differs from unity.

## 3 Applications

### 3.1 Filtering of Data with Peaks

**Figure 4:**
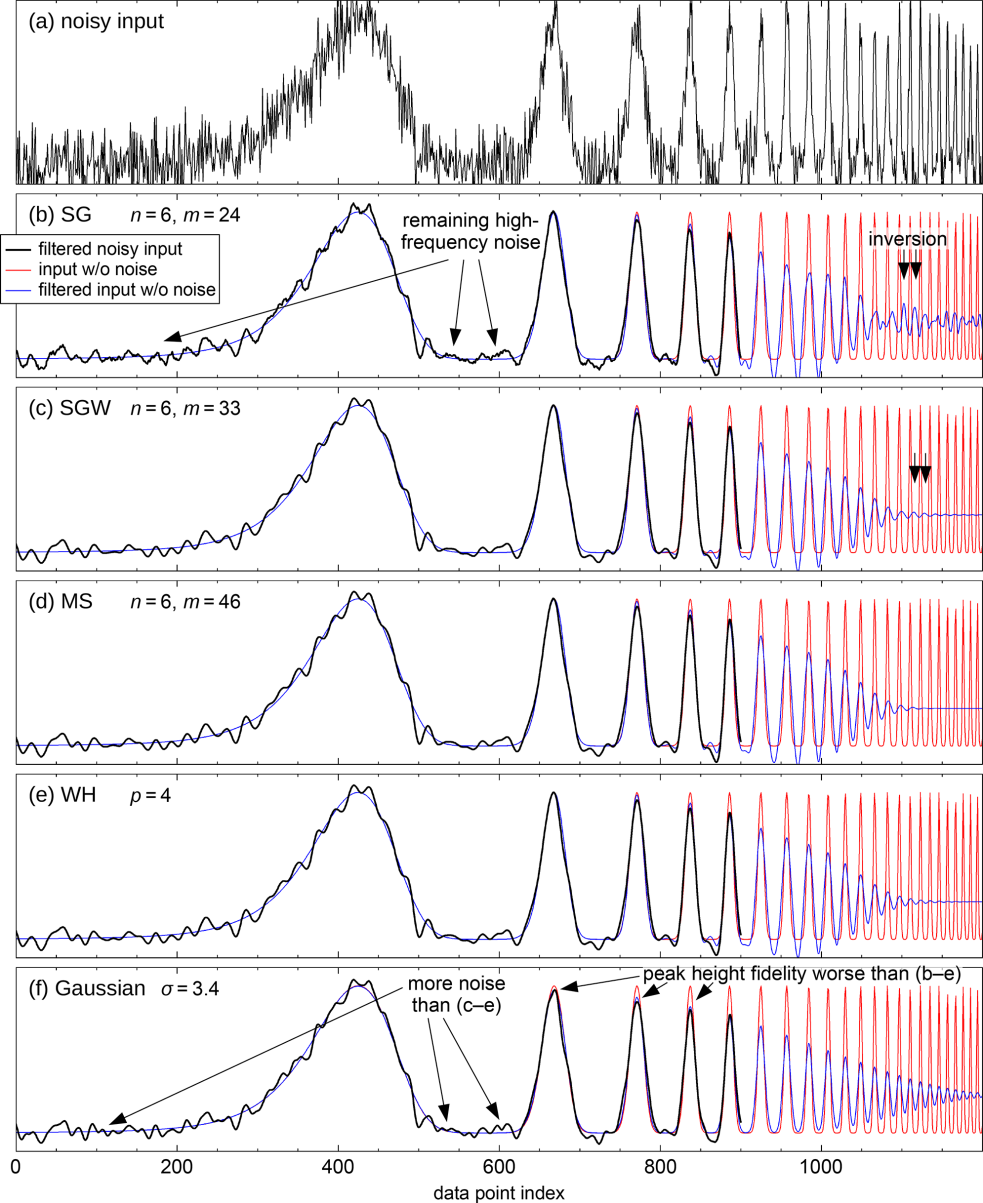
Filtering of a series of Gaussian peaks with successively smaller widths. The input without superimposed noise is red in (b–f); the corresponding filtered curves are blue. The input with added noise is in panel (a), and the corresponding filtered curves are black in (b–f). The smoothing filters applied are (b) traditional Savitzky–Golay, (c) Savitzky–Golay with Hann-square weights, (d) convolution with a modified sinc kernel, (e) Whittaker–Henderson smoothing, and (f) convolution with a Gaussian; all of these have a similar cutoff frequency. The noisy input curve (a) was obtained by adding Gaussian-distributed noise with a standard deviation of 1/8 of the peak height; filtering of the noisy data (black) is shown only up to point 900 to avoid cluttering. Note the poor suppression of high-frequency noise and the phase inversion of the traditional SG filter.

One of the attractive properties of SG filters can be seen in Figure 4, which can be considered an archetypal example of SG filtering.^9^

With SG smoothing and the other filters discussed here, the heights of narrow peaks (in the range of data points 650–950 in this figure) are better preserved than with many other smoothing filters with similar bandwidth, e.g., convolution with a Gaussian. Figure 4b also demonstrates some of the shortcomings of traditional SG filters. Suppression of high-frequency noise (small wiggles) is unsatisfactory; this becomes obvious when comparing the black line in Figure 4b with panels c–e, where the output is much smoother. With high input frequencies (narrow peaks above data point 1050), peak inversion occurs in some regions. This is related to the odd sidelobes of the frequency response (Figure 1b), where the gain has a negative value. This problem has been noted previously, but the solution suggested in that work^31^ was very similar to convolution with a Gaussian, sacrificing the good preservation of peak heights and the flat passband and sharp cutoff in the frequency domain.

Upon visual inspection, the noise suppression of the SGW filter with the Hann-square weight function is almost as good as convolution with the MS kernel and WH smoothing (Figure 4c–e). Nevertheless, in the high-frequency response (above data point 1100), the SGW still shows phase inversion, though with much lower amplitude than the traditional SG filter (arrows). These artifacts come from the sidelobes in the frequency response (Figure 1b); they would be lower when choosing a weight function with better sidelobe suppression, such as the cube of the Hann function (not shown). The MS smoothed signal in Figure 4d shows only weak out-of-phase wiggles above data point 1100; these are mostly ringing artifacts caused by neighboring peaks and related to the sharp frequency cutoff. The WH filter has a more gradual cutoff in the stopband; thus, the ringing remains invisible in this plot. The ringing is comparable for the MS and WH filters (see the inset in Figure 1), but there are no regions where the amplitude of the WH filtered signal sharply drops to zero in Figure 4. The noise suppression of the SGW, MS, and WH filters is better than that of convolution with a Gaussian. At the same time, SGW, MS and WH provide better peak height fidelity below data point 1000.

**Figure 5:**
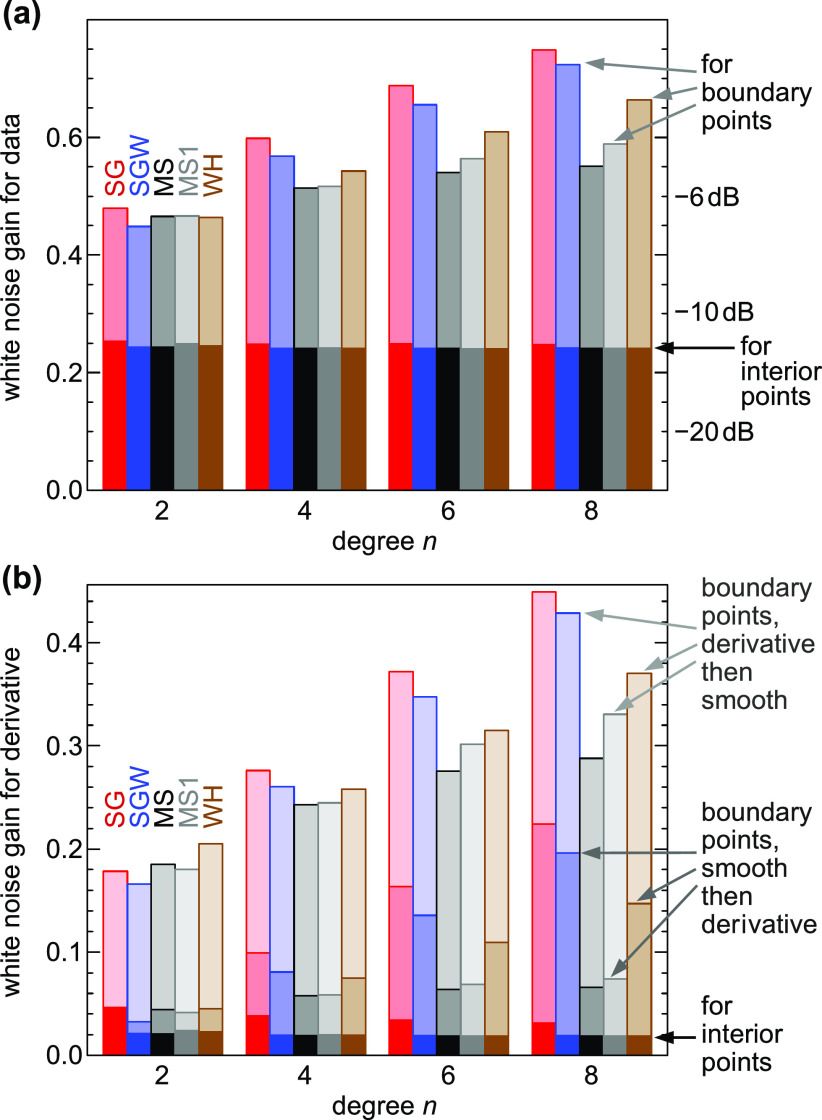
White noise gain of the filters for different degrees *n*. As in Figure 3, the smoothing parameters have been set such that the peak of a Gaussian with fwhm = 20 is attenuated to 90% of its original height. (a) For smoothing of data, the white noise gains are shown for interior points (full bars) and points at the boundary of the data (lighter colors). (b) White noise gains of the derivative calculated numerically. For boundary points, the results strongly depend on the sequence, whether the differentiated data are smoothed (lightest colors) or the smoothed data are differentiated (lower noise). The MS1 kernels are similar to MS and described in the Supporting Information.

Figure 5a provides a comparison of the noise suppression with different smoothing methods and *n* values. This figure was calculated for a Gaussian with fwhm = 20 and 90% peak height fidelity, which is the setup as in Figure 3. The noise gains, i.e., the ratio between the output and input root-mean-square noise, were calculated for white noise. For interior points (full bars), all filter types yield similar results, with the noise of the traditional SG filters above that of the others. Only for *n* = 2, the MS1 kernel provides slightly less noise suppression than SGW, MS, and WH because its cutoff in the frequency domain is less sharp. Comparing different filter degrees, *n* = 4 shows a small improvement (reduced noise gain) over *n* = 2, but there is no further improvement for higher *n*. At the boundary points, the noise is 2–3 times higher and increases with *n*. For *n* ≥ 4, our linear extrapolation method followed by convolution with the MS kernel provides the lowest noise.

**Figure 6:**
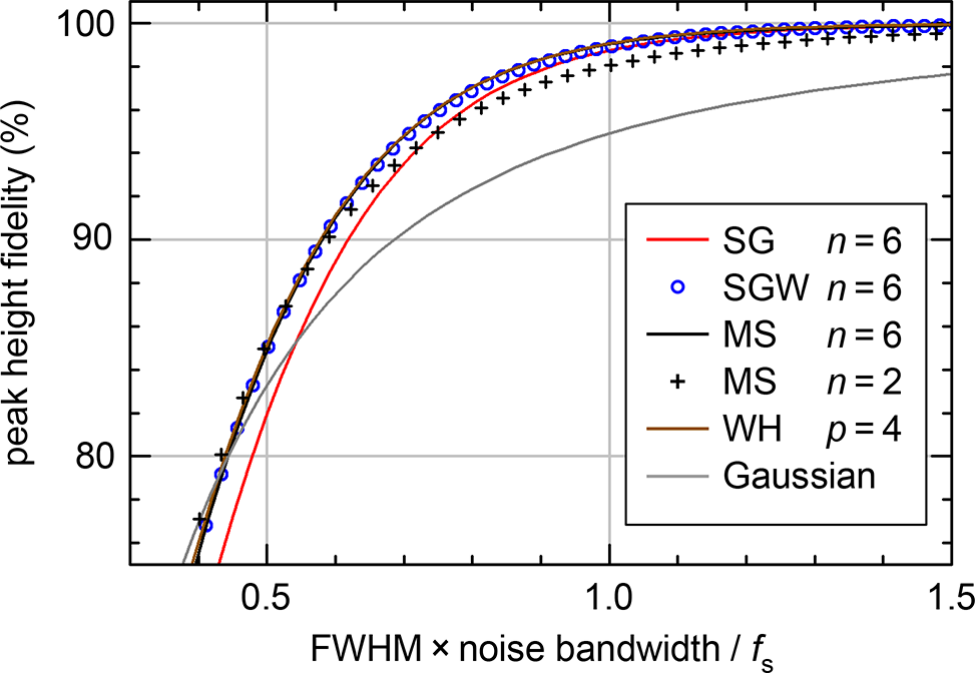
Impact of filtering on the height of a Gaussian peak with a given full width at half-maximum (fwhm). For a given peak height fidelity (i.e., relative peak height after filtering), the classical SG filter and convolution with a Gaussian (except for strong attenuation of the input) require a larger noise bandwidth, i.e., weaker suppression of noise. The curves for the *n* = 6 MS and *p* = 4 WH smoothers are almost identical and cannot be distinguished in the plot. The calculations were done with the filters of Figure 1, but the results normalized as in this plot depend only weakly on the extent of smoothing (the *m* or λ value).

Figure 6 provides information beyond the test case of smoothing an fwhm = 20 peak with 90% peak height fidelity. As a measure of white noise gain, we define the noise bandwidth as the integral over the power spectrum of the kernel, with the full bandwidth corresponding to the Nyquist frequency *f*_s_/2. Then, the white noise gain is proportional to the square root of this bandwidth. Increasing the bandwidth causes less attenuation of a peak with a given fwhm; sharper peaks (lower fwhm) require higher bandwidth. If we take the product of the noise bandwidth and the fwhm as the abscissa, we can plot the peak height fidelity as a function of this product, largely independent of the specific bandwidth or fwhm value. Figure 6 then provides a figure of merit of the various filters: If a given peak height fidelity is required (e.g., 90% of the original height), the leftmost curve has the lowest noise bandwidth for white noise; that is, it best suppresses the noise. The gray curve shows that convolution with a Gaussian kernel performs worst (except when the peaks are strongly attenuated to less than 80% of their original height). The MS and WH filters are almost equal (the difference is less than the line width in Figure 6) and best, and the SGW comes very close. The traditional SG filter is worse than our filters because of the poor attenuation of high-frequency noise (see above). For MS filters with *n* = 6 (or WH with the corresponding *p* = 4), improving the peak height fidelity from 90 to 99% requires increasing the bandwidth by a factor of 1.7, which corresponds to a increase in white noise gain.

As shown in Figure 5a, the noise suppression of *n* = 2 filters is slightly weaker than that of higher degrees *n*, due to the more gradual cutoff in the frequency domain. The “+” symbols in Figure 6 show that this is more pronounced if very high fidelity of the peak heights is desired. For 90% peak height fidelity (the test case of Figure 5), there is no advantage in using higher degrees than *n* = 4 (or the corresponding *p* = 3 for WH); that is, there is no benefit of a steeper cutoff in the frequency domain. For interior points, the white noise gain does not decrease for *n* > 4, whereas the noise at the boundaries increases, and the range where artifacts occur also grows. If a higher fidelity is required, such as preserving the height of a given Gaussian with 99% (rather than 90%) fidelity, increasing *n* from 4 to 6 would provide a small decrease of the white noise gain for interior points (by ≈3% for MS and WH). Still, the noise at the boundaries would increase by ≈3 and 8% for MS and WH, respectively. We do not see any value in using higher degrees than *n* = 6 (*p* = 4 for WH) for signals that consist of Gaussian peaks. Higher degrees are justified only for signals that inherently have a very sharp cutoff in the frequency domain. In that case, a similarly sharp cutoff of the filter could better separate signal and noise, but the handling of near-boundary data becomes more problematic.

For practical use of the MS or SGW filters, the user has to determine the half-width *m* of the kernel required to preserve the peak height with a given fidelity. This has been done previously for the traditional SG filters;^32,33^ here, we present how this can be accomplished for the SGW, WH, and MS filters. For degrees 2 ≤ *n* ≤ 10, we find that the *m* value for a given peak height attenuation can be expressed as
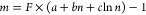
19where *m* should be rounded to the nearest integer. Here, *F* is the full width at half-maximum of the peak. The coefficients *a*–*c* are given in Table 2 for peak height fidelity values of 90, 95, 98, and 99%.

**Table 2:** Parameters for Equations 19 and 20^a^

method	fidelity (%)	*a*	*b*	*c*
SGW	90	0.8512	0.2565	0.1999
	95	0.5993	0.2163	0.1924
	98	0.3769	0.1850	0.1789
	99	0.2578	0.1701	0.1643
MS	90	1.2354	0.4060	0.1015
	95	0.8874	0.3402	0.1290
	98	0.5739	0.2881	0.1495
	99	0.4013	0.2642	0.1470
WH	90	1.7168	–0.0173	0.0697
	95	1.2647	–0.0243	0.1687
	98	0.8842	–0.0286	0.2471
	99	0.6827	–0.0296	0.2842

a These parameters are used for calculating the filter parameters that result in a decrease of the peak height of a Gaussian peak to 90, 95, 98, and 99% of its original value. The table is valid for *m* ≥ *n*/2 + 2 in the case of the MS filters and *m* ≥ *n*/2 + 1 for SGW.

For WH smoothing, a similar equation can be used:
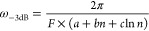
20with *n* = 2(*p* – 1) and the WH parameters (from a least-squares fit for 2 ≤ *p* ≤ 5) in Table 2. Then eq 12 can be used to determine the λ smoothing parameter.

### 3.2 Smoothing for Calculating the Derivatives

Smoothing is often applied when the data are to be differentiated. Differentiation amplifies the high-frequency components, and one of the main applications of SG filtering lies in smoothing followed by taking the derivative.^4^ Since SG filters are based on a polynomial fit, the derivative can be calculated analytically, making SG filtering popular for this application. It has been noted previously^12^ that essentially the same can be accomplished by SG filtering followed by numeric differentiation, with a slight improvement of noise suppression when calculating the derivative of the filtered data *z* as (*z*_*i*+1_ – *z*_*i*–1_)/2. The slightly improved noise suppression is mainly due to the difference between the frequency response of analytic differentiation (multiplication with ω) and that of the numerical derivative (multiplication with sin ω when taking the sampling frequency as *f*_s_ = 1). The latter reduces the effect of the high-frequency sidelobes of the SG filter (at the Nyquist frequency, sin ω = sin π = 0). In the present work, we use numerical differentiation by simply taking *z*_*i*+1_ – *z*_*i*_, which is closer to analytic differentiation than the method mentioned above. This numerical method may be unsuitable for some applications because it causes a shift by 1/2 data point; then the above method^12^ must be used. For most purposes, the difference between the differentiation methods is irrelevant (except for differences in the noise suppression of the traditional SG filters).

Figure 5b compares the noise gain of the different smoothing methods with this setup and for filter parameters leading to a peak height fidelity of 90% at fwhm = 20. For interior points (full bars), the noise suppression of the filters with good stopband suppression is better by a factor of 2 compared with that of the traditional SG. For lower bandwidth (stronger smoothing, as permitted for larger fwhm), the difference would be even more pronounced. As an example of filtering and differentiating real experimental data, Figure S7 demonstrates the poor noise suppression of the SG filter for smoothing an infrared spectrum; it also shows that SG filtering performs even worse when higher derivatives should be calculated.

**Figure 7:**
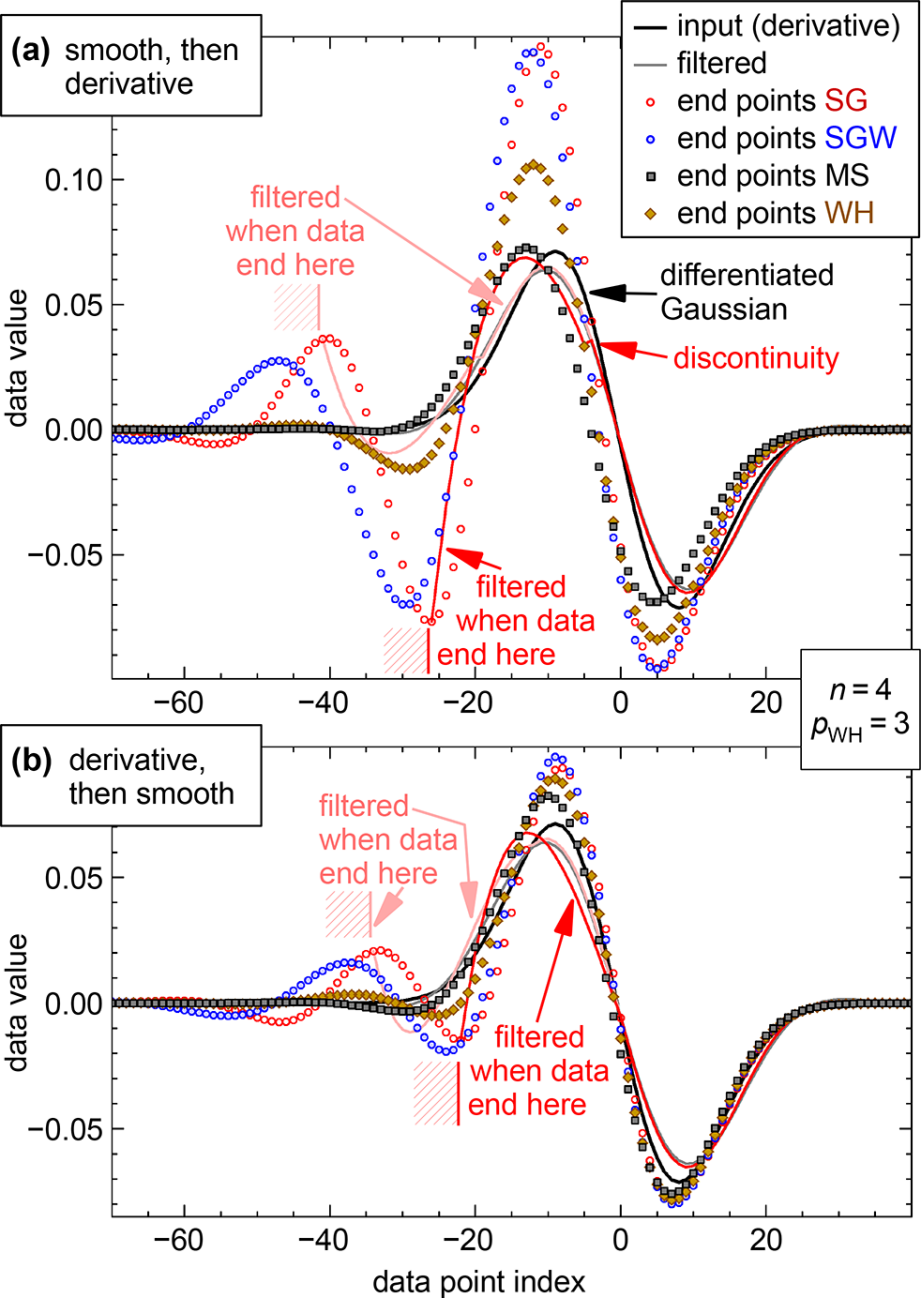
Filtering and differentiation of a Gaussian peak near a boundary. (a) Filtering of a Gaussian (fwhm = 20), followed by numeric differentiation. The filter parameters were set for a peak height fidelity of 95% for the Gaussian. This leads to attenuation of the peaks of the derivative to ≈90% (gray curve, mostly hidden by the pink curve). When the left boundary of the input data is at the position marked by the red or pink shaded area, filtering with a traditional SG filter and differentiation leads to the red and pink curve, respectively. The end points of the filtered and differentiated curves for all positions of the boundary are shown as red circles for the SG filter and in other colors for the other filters. (b) Same as in (a) but with first differentiating followed by smoothing with the same parameters as in (a). All data for filters of degree *n* = 4 (corresponding to *p* = 3 for WH). Kernel half-widths *m* are 22, 34, and 38 for SG, SGW, and MS, respectively.

For interior points, filtering and (numerical) differentiation are convolution operations, thus commutative, and the result does not depend on which of these operations is performed first. This is not the case for near-boundary points. For analysis of the near-boundary behavior, we use essentially the same test case as in section 2.6, a Gaussian peak near or at the boundary of the data. As differentiation enhances high-frequency components, which are selectively attenuated by the filters, we now set the filter parameters such that filtering reduces the height of the Gaussian to 95% of its original value, not 90%. This attenuates the peaks of the derivative to about 90%. Results of filtering and differentiating a near-boundary Gaussian (fwhm = 20) are shown in Figure 7.

The red curve in Figure 7a shows the result of smoothing with the traditional SG filter if the boundary of the input data is at −26. At the boundary, the deviation between the smoothed and differentiated curve (red) and the derivative of the Gaussian (black) must be considered unacceptable. The same is true when the left boundary is at −41 (pink curve). SG filtering followed by numerical differentiation can also show a discontinuity at a distance of *m* from the boundary (red arrow). This discontinuity would disappear when using analytic differentiation, but then the deviations from the differentiated Gaussian are even slightly larger at the end points. As in Figure 3, the red circles show the end points of the smoothed and differentiated data for all positions of the boundary. Obviously, for traditional SG filtering, these end points are far from the expected values for many positions of the boundary, not only for the extreme cases marked by the red and pink curves. Figure 7a shows that SGW is hardly any better. Therefore, we consider both the SG and SGW filters unusable for smoothing and differentiating near the boundaries. WH is clearly better, and the best behavior is observed for MS convolution with linear extrapolation of the data (dark gray squares). Yet also here, the end points deviate from the derivative of the input.

Figure 7b shows the alternative sequence. First taking the derivative and then smoothing yields better fidelity of the processed curves. SG filtering again performs worst, closely followed by SGW, and MS performs best. At first glance, this sequence may seem preferable; the smoothed curves preserve the position of the zero derivative at the peak maximum before differentiation. However, as expected from the usual trade-off between fidelity and noise suppression, the price to pay for the better near-boundary performance of taking the derivative first is much poorer noise suppression at the boundaries (see Figure 5b). For the WH smoother, the reason is obvious: with a penalty on the second derivative (*p* = 2), the second derivative of the smoothed data at the boundary will be very low. There are no input data at the outside that would demand a nonzero value of the second derivative (calculating the second derivative requires data left and right of that point). After differentiation, a penalty on the second derivative corresponds to a penalty on the first derivative, which limits the data values at the boundary (red curves in Figure S6). When first taking the derivative, then differentiating, the penalty is on the second derivative, and the first derivative is unconstrained, leading to overshoot at the boundaries (cyan curves in Figure S6). Essentially the same is true for higher degrees: a constraint on a lower derivative means a stronger limitation for the overshoot at the boundaries. For SG-based filters, the explanation is essentially the same: the tendency to overshoot at the boundaries increases with the degree of polynomials in a polynomial fit. Differentiation of the filtered data is like lowering the degree of the polynomial fit.

The increase of noise when first taking the derivative, then smoothing, depends on the cutoff frequency of the filters. For weak smoothing (e.g., for preserving narrow peaks; peak height fidelity 90% for fwhm = 5), the increase of the noise is less than a factor of 2 (but the noise gain at the boundary is still a factor of ≈5 above the noise gain for the interior). For strong smoothing, the discrepancy between the differentiation first and smoothing first increases. This means that the “derivative first, then smoothing” method is not useful for strong smoothing because of insufficient noise suppression near the boundaries. The near-boundary artifacts of MS smoothing followed by numerical differentiation (Figure 7a) are the lesser evil.

The trade-off between fidelity and noise suppression does not necessarily apply when comparing different smoothing methods. When smoothing first, then taking the derivative, Figure 7a shows that the MS method clearly provides the best fidelity at the end points; at the same time, for *n* ≥ 4, this method has the best noise suppression of all (see Figure 5b). On the other hand, the noise suppression of the traditional SG filter is worst for both interior points and at the boundaries in all cases except one (*n* = 2 and “derivative then smooth”); nevertheless, its filtered curves show the worst artifacts near the boundaries. Thus, there are methods that perform better when smoothing near-boundary data, in terms of both artifacts and noise suppression, and others that are worse. The traditional SG filter is clearly worst, and for *n* ≥ 4, the MS filter performs best. At *n* = 2, MS and WH are almost equal, with MS providing only slightly better noise for the derivatives. In this case, the choice may depend on convenience of implementation.

## 4 Conclusions

In summary, we have presented and analyzed three types of smoothing filters superior to traditional SG smoothing. All of these filters provide much better suppression of high frequencies, which is especially important if the derivative of the data is of interest. Adding weights to the polynomial fit in SG filtering (SGW) leads to a substantial improvement. Still, the two other methods discussed here, convolution with a modified sinc kernel and Whittaker–Henderson smoothing, outperform the SGW filters. For the interior of the data, the frequency response and noise suppression of the MS and WH filters are similar. WH smoothing handles near-boundary values in a natural way. Nevertheless, MS convolution combined with linear extrapolation of the data provides substantially better results near the boundaries for degrees *n* ≥ 4, that is, fewer artifacts and better noise suppression for the derivative. MS convolution also has the advantage of being numerically more stable than the WH method, irrespective of the smoothing parameters (at the expense of higher computing time for MS convolution with large kernels). The WH method is superior to traditional SG smoothing and was named “a perfect smoother”.^15^ Our analysis shows that improvements beyond WH smoothing are possible, and we consider convolution with the MS kernels, together with linear extrapolation at the boundaries, the best method currently available.

## Supporting Information Available

Figures S1–S9, details on the MS1 smoothing kernels, the SGW weight function for near-boundary points, and smoothing of data with Lorentzian peaks (PDF)
Java code for SGW smoothing, MS and MS1 smoothing, and WH smoothing; GNU Octave code for MS and MS1 smoothing (ZIP)
