# Correction to “Why and How Savitzky–Golay Filters Should Be Replaced”

**DOI:** 10.1021/acsmeasuresciau.3c00017

**Published:** 2023-05-04

**Authors:** Michael Schmid, David Rath, Ulrike Diebold

In the Supporting Information ZIP folder, in the file named “WeightedSavitzkyGolaySmoother.java”, the function savitzkyGolayBandwidth() is incorrect. It is correct in the two other java files. The Supporting Information for this correction notice provides the corrected computer code. The authors would like to thank Marios Chatzikos for pointing out the error.

## Supporting Information Available

Java code for SGW smoothing, MS and MS1 smoothing, and WH smoothing; GNU Octave code for MS and MS1 smoothing (ZIP)

