# Supplementary material for: Why and How Savitzky–Golay Filters Should Be Replaced
Source: ACS Meas Sci Au. 2022 Feb 18;2(2):185–96. doi: 10.1021/acsmeasuresciau.1c00054 (PMC9026279; doi:10.1021/acsmeasuresciau.1c00054)
Supplement: Supplementary file 1 — tg1c00054_si_001.pdf [file tg1c00054_si_001.pdf]

Why and how Savitzky-Golay filters should be replaced

Michael Schmid,* David Rath, and Ulrike Diebold

Institute of Applied Physics, TU Wien, 1040 Vienna, Austria

E-mail: schmid@iap.tuwien.ac.at

ACS Measurement Science Au (2022)

DOI: 10.1021/acsmeasuresciau.1c00054

Supporting Information

Figure S1: Kernels of the different filter types

Figure S2: Frequency response of the different filter types

Figure S3: Frequency response vs. *m*

Figure S4: Construction of the MS1 and MS window function from Gaussians

Figure S5: End points when smoothing a near-boundary Gaussian peak

Figure S6: Impact of the sequence of smoothing and differentiation

Figure S7: Smoothing of an experimental FTIR spectrum of polystyrene

The MS1 smoothing kernels

The SGW weight function for near-boundary points

Smoothing of data with Lorentzian peaks

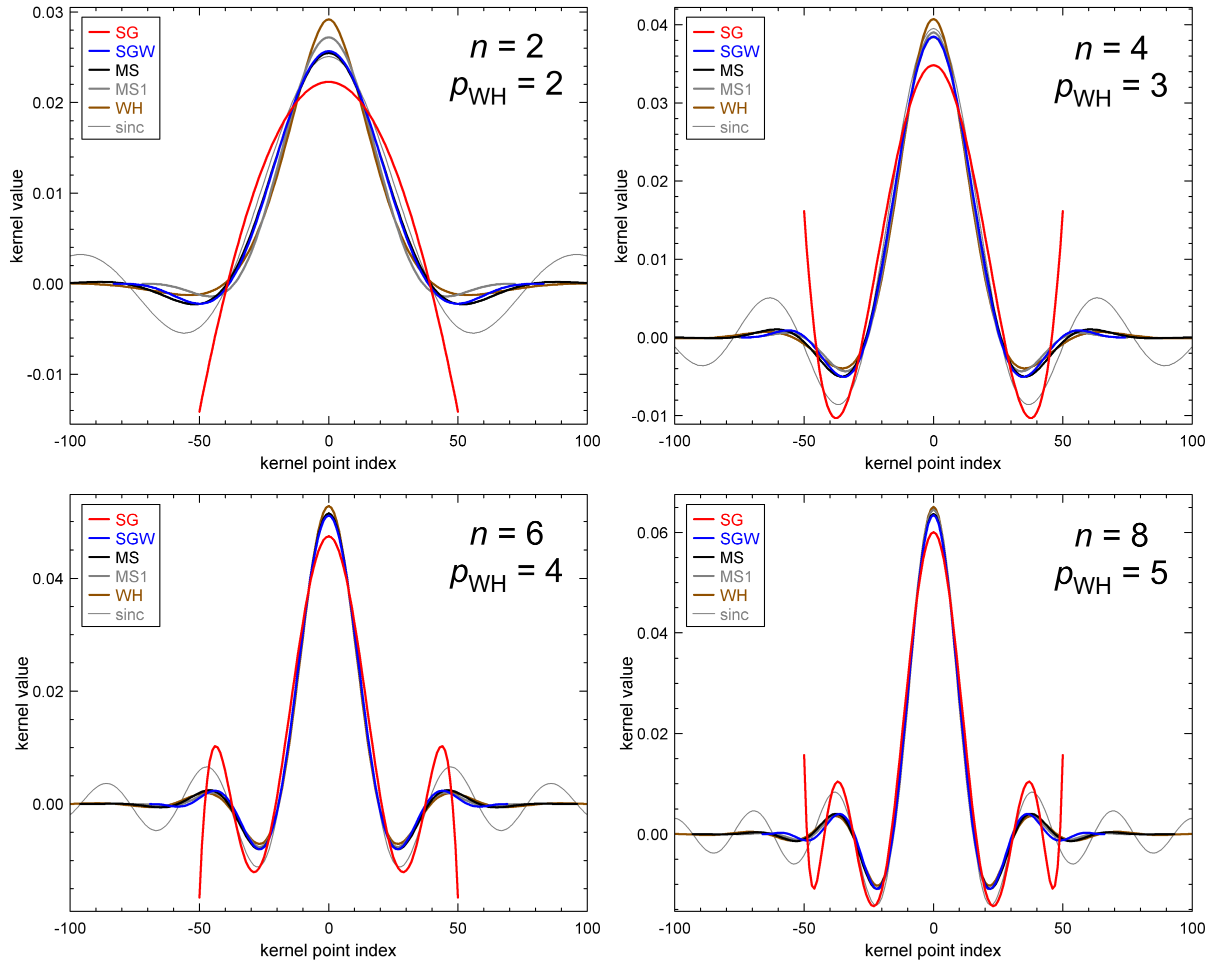

Figure S1: **Kernels of the different filter types.** Comparison of the filter kernels for different degrees n. The SG kernels have a half-width of $m = 50$. For the other filters, the m values (or λ for the WH) were chosen to yield a comparable cutoff frequency. For high degrees n, the kernels of the MS, MS1, and WH filters are very similar, thus the curves are almost exactly on top of each other. The MS-type kernels are based on the sinc functions shown as thin lines.

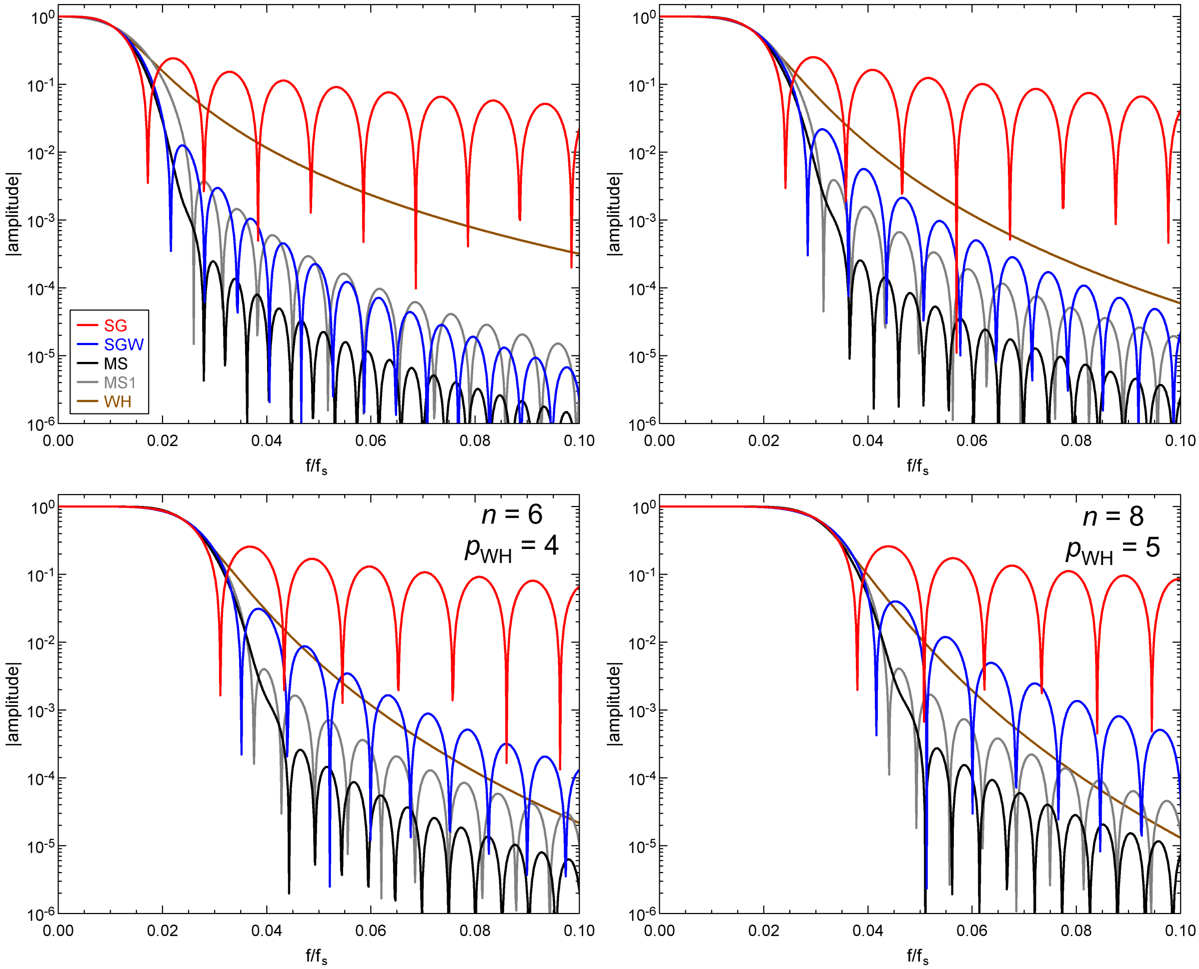

Figure S2: **Frequency response of the different filter types.** Comparison of the filters for different degrees n. The SG kernels have a half-width of $m = 50$. For the other filters, the m values (or λ for the WH) were chosen to yield a comparable cutoff frequency. Note that the MS1 filters have a softer cutoff than the other filters at $n = 2$ (gray arrows).

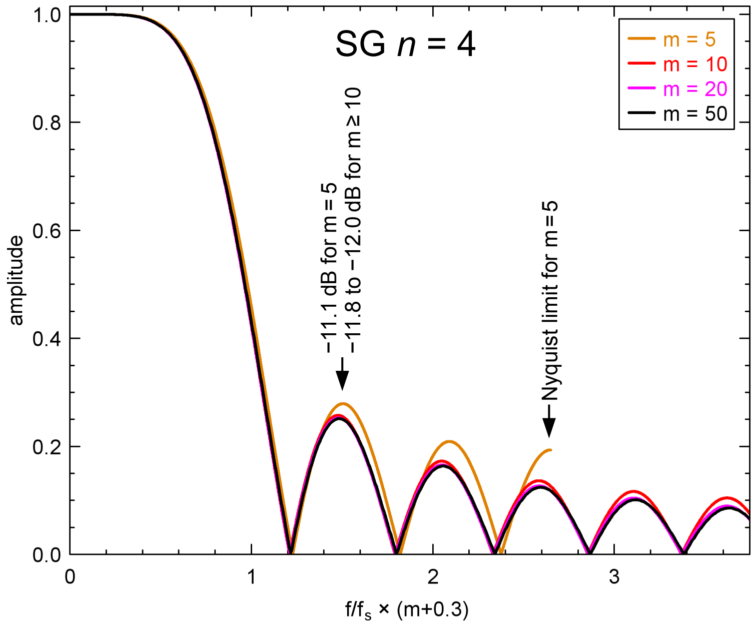

Figure S3: **Frequency response vs. *m*.** Comparison of the frequency response of a traditional SG filter of degree 4 for different values of the kernel halfwidth m. The frequency axis has been scaled such that the -3 dB points approximately coincide. [For a more accurate scaling, see equation (14) of the main text]. For the other filter types, the response curves depend much less on the m value when the frequency axis is appropriately scaled; there is only a small increase of the amplitude close to the Nyquist limit (not shown).

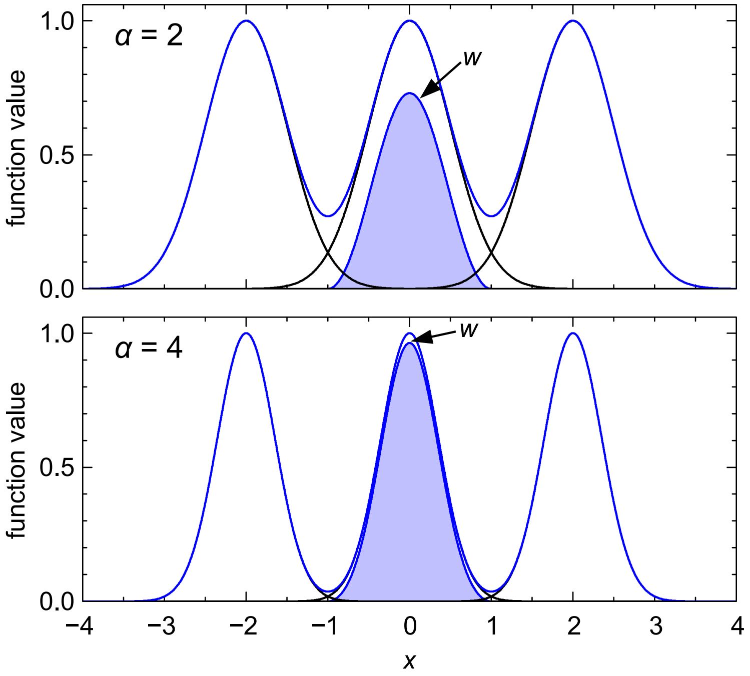

Figure S4: **Construction of the MS1 and MS window function from Gaussians.** Adding three Gaussians (black lines, mostly hidden behind the blue lines) according to equation (4) of the main text results in a curve with essentially zero derivative at $x = \pm 1$ (blue line). The window function w (shaded blue), defined between $x = -1$ and $+1$ is then created by subtracting a constant. Since the kernel must be normalized anyhow, there is no need for normalization of the window function (its peak value and its integral can have arbitrary values). The top frame shows the window function of the MS1 kernels, with $\alpha = 2$; the bottom frame that of the MS kernels ($\alpha = 4$).

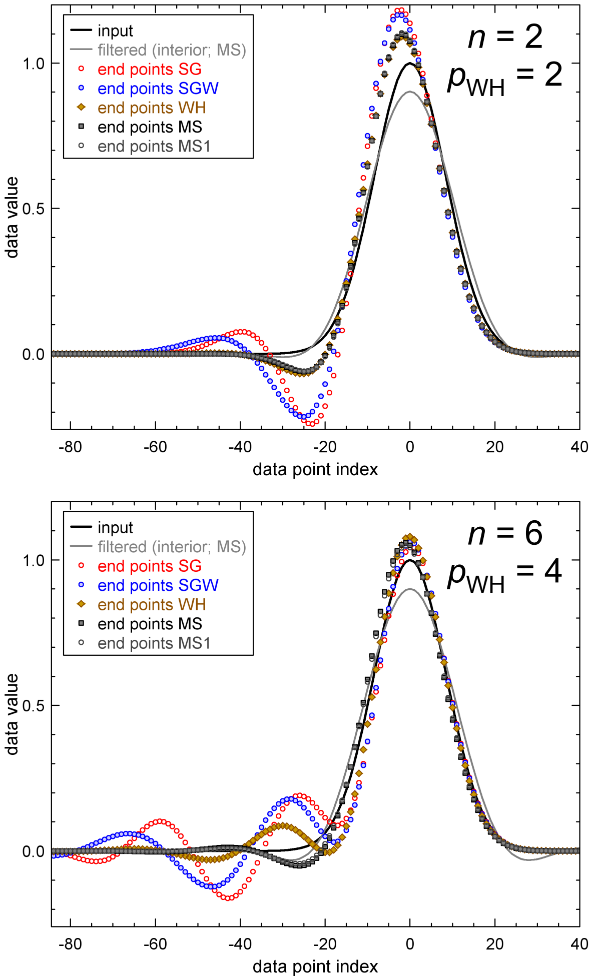

Figure S5: **End points when smoothing a near-boundary Gaussian peak.** The figures show the filtered data points at the boundary for different positions of the boundary of the input data, as in Fig. 3c of the main paper, but also for different degrees n. The near-boundary behavior of the MS and MS1 kernels is very similar (points overlap) because both are based on linear extrapolation of the data before filtering.

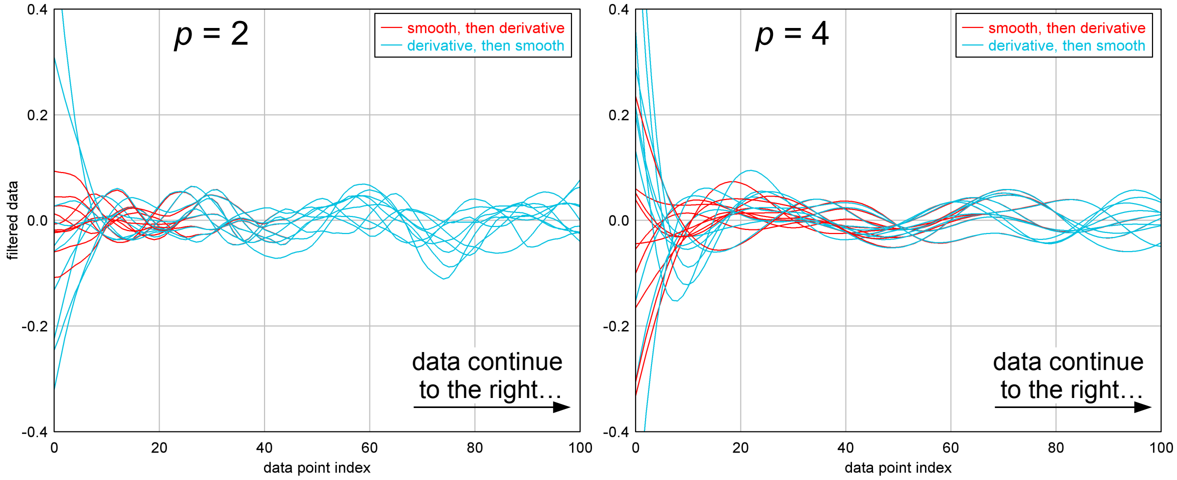

Figure S6: **Impact of the sequence of smoothing and differentiating on near-boundary points.** Each figure shows the result of filtering 10 different data sets with white noise with a standard deviation of unity. The boundary of the input data is at the left. The red curves show the result of first smoothing, then taking the derivative, and the cyan curves are for numerical differentiation followed by smoothing. In both cases, a WH smoother has been used, with the smoothing parameter λ chosen such that the peak height of a Gaussian with FWHM $= 20$ is attenuated to 90% of its original value. The panels differ by the penalty degree p of the smoother; higher p leads to more noise at the boundary. Note that the red and cyan curves merge towards the right, because the sequence of smoothing and differentiating plays no role for interior points.

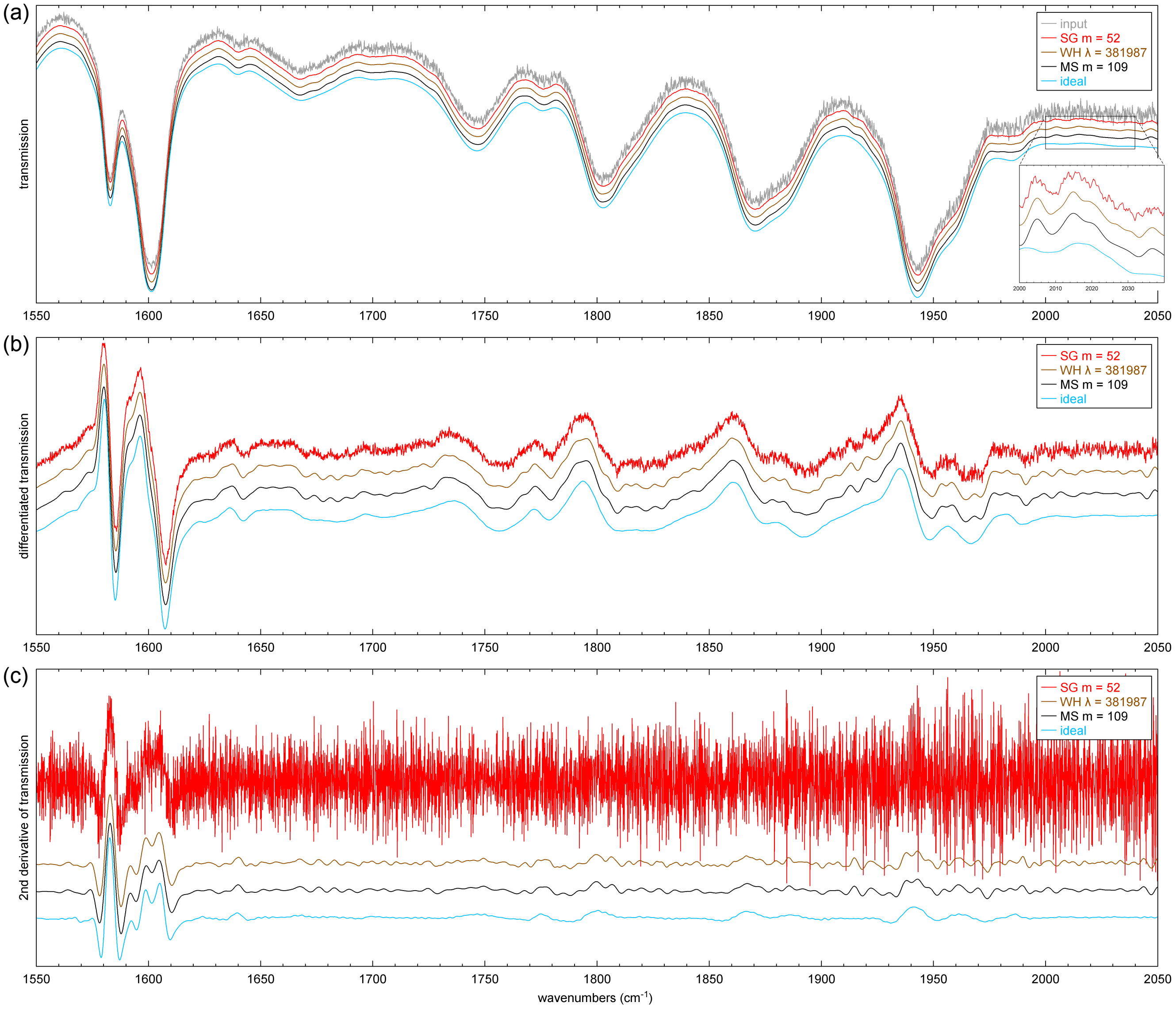

Figure S7: **Smoothing of an experimental Fourier-transform infrared spectrum of polystyrene.** **(a)** The noisy input spectrum (gray), with a data point spacing of $0.12\,\mathrm{cm}^{-1}$, was smoothed by $n = 4$ ($p_{\mathrm{WH}} = 3$) filters selected such that the absorption peak at $1583\,\mathrm{cm}^{-1}$ with an estimated FWHM of $8\,\mathrm{cm}^{-1}$ is attenuated to $\approx 99\%$ of its original height. Due to the good data quality, upon visual inspection, the smoothed curves appear almost identical to the light-blue curve named "ideal", which was obtained by averaging over 1024 such spectra and slight smoothing to eliminate a small signal contribution with a periodicity of $0.4\,\mathrm{cm}^{-1}$ (possibly caused by optical interference fringes). Higher magnification reveals residual noise in the SG-filtered curve (inset). **(b)** When differentiating the smoothed spectra, which is often done to remove the uneven "background" before comparing or normalizing spectra, it becomes obvious that the noise suppression of the traditional SG filter is much worse than that of the other filters. **(c)** The second derivative of the SG-smoothed curve is dominated by noise, while the other smoothing methods clearly show the peaks of the spectrum in the 2nd derivative. SGW filtering (not shown) is visually indistinguishable from MS. The small wiggles in the differentiated curves (b, c), e.g. around $1680\,\mathrm{cm}^{-1}$, have a periodicity of $\approx 7\,\mathrm{cm}^{-1}$ or more, which places them inside the signal bandwidth (though close to the cutoff). Thus they cannot be suppressed by low-pass filtering without affecting the peak heights. Curves are offset vertically for clarity.

The MS1 smoothing kernels

The MS1 smoothing kernels are similar to the MS kernels, but with a smaller kernel size. The MS1 kernels are based on a window function reaching out one half-wave of the sinc function less than the MS kernel. Then the kernel of degree n has n inner zeros, the same as the SG and SGW kernels. We chose $\alpha = 2$ for the window function (4), which yields a frequency cutoff similar to that of the other non-MS filters except for $n = 2$, where the cutoff is more gradual (Fig. S2). With $\alpha = 2$, the Gaussian window function becomes similar to the Hann (cosine-square) window, but yields slightly better sidelobe suppression. Similar to (7), the kernel is given by

$$a_i = A \times w_\alpha(x) \times \left[\sin\left(\frac{n+2}{2}\pi x\right) / \left(\frac{n+2}{2}\pi x\right) + \sum_{j=0}^{n/2-2} \kappa_j^{(n)} x \sin((j+1)\pi x) \right] . \tag{S1}$$

Without the correction terms with κ, the overshoot in the passband is of the MS1 kernels is higher than for the MS kernels, which is understandable due to the fact that the overshoot stems from windowing and truncating the sinc function, which is more severe for the MS1 kernels. Without correction, we find $\approx 1.7\%$ (0.15 dB) overshoot for $n = 4$ and some waviness with up to $\approx 1\%$ overshoot for $n \geq 6$. As for the MS kernels, the correction coefficients $\kappa_j^{(n)}$ are given by (8), but with different $a_j^{(n)}$, $b_j^{(n)}$, and $c_j^{(n)}$ values, provided in Table S1. With these coefficients, the overshoot is below 2×10^{-4} (0.002 dB).

Table S1: Coefficients for (8), which yields the $\kappa_j^{(n)}$ values in (S1), to ensure a flat passband of the MS1 kernels. For $n = 2$, no such correction is needed.

n	4	6	6	8	8	8	10	10	10	10
j	0	0	1	0	1	2	0	1	2	3
$a_j^{(n)}$	0.02194	0.00190	0.02306	0.00659	0.00232	0.02105	0.00097	0.00898	0.00242	0.01919
$b_j^{(n)}$	0.05028	0.00848	0.13048	0.05793	0.01030	0.16647	0.00207	0.09902	0.01006	0.18954
$c_j^{(n)}$	0.76562	1.26250	1.22656	1.91562	2.27266	1.98125	3.74375	2.70781	3.29688	2.78496

The frequency cutoff of the MS1 kernels is comparable to that of the other filtering methods, with the exception of $n = 2$, where the MS1 cutoff is less steep. In the stopband, the MS1 filters have less sidelobe suppression than the MS kernels, but they are still superior to all the other smoothing methods discussed here, see Fig. S2. The first sidelobes are at ≈ -48 dB and as for the MS kernels the decrease towards higher sidelobes is $1/f^4$ (80 dB/decade). For most practical applications, reducing the sidelobes to a level below that of the SGW and MS1 filters will have hardly any effect on the smoothed data; the advantage of the MS filters lies in the sharper cutoff for $n = 2$ and the easier implementation for $n = 4$ (no correction terms).

For a given bandwidth (e.g. for replacing a traditional SG filter), in analogy to (16), the kernel halfwidth m can be determined by

$$m_{\mathrm{MS}} = (0.270 + 0.249n)/b_{-3\,\mathrm{dB}} - 1 . \tag{S2}$$

For handling near-boundary data, we use essentially the same type of weighted linear extrapolation as for the standard MS kernels:

$$w_{\text{fit}}(i) = \cos^2 \frac{\pi i(n+2)}{4\beta(m+1)} \tag{S3}$$

if the argument of the cos function is less than $\pi/2$; otherwise $w_{\text{fit}}(i) = 0$. As for the MS filters, this weight function ends near the first zero of the sinc for $\beta = 1$. To ensure noise suppression at the boundaries comparable with (or slightly better than) the best non-MS filters, we use

$$\beta = 0.65 + 0.35\exp(-0.55(n-4)) \tag{S4}$$

With this choice of β, artifacts when smoothing a near-boundary peak are very similar to the standard MS smoother, i..e., much less than those of the SG or SGW filters, and also less than those of the WH smoothers for $n \geq 4$.

For smoothing data with Gaussian peaks, the kernel halfwidth m for obtaining a given peak height fidelity can be determined from (19) with the coefficients in table S2.

Table S2: Parameters for (19), for calculating the MS1 kernel halfwidth m that results in a decrease of the peak height of a Gaussian peak to 90, 97 and 99% of its original value.

method	fidelity (%)	a	b	c
MS1	90	0.3248	0.4024	0.1658
	95	0.0792	0.3336	0.2227
	98	−0.1516	0.2791	0.2704
	99	−0.2726	0.2542	0.2799

The SGW weight function for near-boundary points

The SGW filter is based on a polynomial fit with a weight function decaying towards zero at the edge of the kernel; the filters discussed in the present work use the Hann-square function for the weights. As mentioned in the main text, keeping the same weight function centered at the output point (the point for which we want to calculate the smoothed value) would lead to fitting the polynomial to a smaller number of points for points near the boundaries (the points outside the data range would be missing), and therefore increase the noise. We therefore modify the weight function by expanding it horizontally (i.e., introducing a scale factor s) such that its sum over the range of valid data remains the same, see Fig. S8. For most weight functions, the scale factor cannot be calculated analytically. Based on numeric evaluation, we found that a function of the form

$$s = 1 - \frac{a}{1 + b\,(m/j)^c} \tag{S5}$$

provides an excellent fit to the numeric results ($R^2 > 0.9999$). Here, j is the number of "missing" data points, i.e., there are $m - j$ data points between the central point (the output point, for which we want to determine the smoothed value) and the end (including the end point, but not the output point). Since we use $c > 0$, the functional form (S5) ensures that the scale factor s becomes unity in the limit $j \to 0$, i.e., for interior points, where all data in the $2m+1$ neighborhood are available.

The fit parameters a, b, and c determined for $m = 1000$ (i.e., essentially in the continuum limit) are given in table S3. Since the exact value of the scale factor s is not critical, we use these parameters independent of the m value. In principle, the same procedure can be used for the traditional SG filters (i.e., a rectangular weight function), where we would achieve a fixed width of the rectangular weight function (and, hence, the same sum over all weight values) with $a = b = c = 1$, leading to $s = m/(j+m)$.

For SGW filtering of near-boundary points, equation (5) must then be modified to

$$x = s\,i/(m+1)\ , \tag{S6}$$

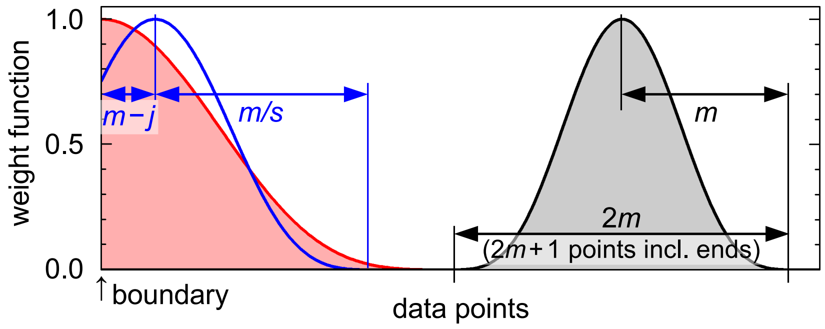

Figure S8: **Scaling the SGW weight function at boundaries.** The black curve shows the Hann-square weight function used in the SGW polynomial fit for interior points. Near the boundary, where data points are missing, the curve is scaled such that the integral (area below the curve) remains the same, see the red and blue curves.

Table S3: Parameters for calculating the near-end scale factor s for the kernel weight function according to equation (S5). For all evaluations in the current work, the Hann-square window function was used.

Weight function	a	b	c
Gaussian-like (4) $\alpha = 2$	0.68096	0.36358	3.68528
Hann	0.67574	0.35440	3.61580
Hann-square	0.63944	0.28417	5.50800
Hann-cube	0.62303	0.25310	7.07317

where i is the distance from the output point (peak of the weight function), measured in data point spacings. The x value is then the argument for the window function, as long as $|x| < 1$. Since the scale factor s is a function of j, the width of the window function (and the kernel) will depend on j, see figure S8. Except for the rectangular weight function of the traditional SG filters, the weight function and the kernel for near-end points will contain fewer than $2m+1$ points.

Since the kernels for the left and right end of the data set are related by mirroring, we have to calculate $m+1$ different kernels (including the "usual" kernel for the interior of the data). The procedure for calculating these kernels is the same as presented in section 2.1 of the main text, see eqns.(1) and (2).

Smoothing of data with Lorentzian peaks

In spectroscopy, peaks with Lorentzian shape can occur if the peak width is determined by the intrinsic line width. This shape is given by

$$y = \frac{1}{1 + 4(x - x_0)^2 / F^2} \tag{S7}$$

where F is the full width at half maximum and x_0 the position of the peak. Compared with a Gaussian, the peaks are sharper at the top, and the tails extend further out (slower decay). In analogy to section 3.1 of the main text, we have determined the smoothing parameters required for Lorentzian peaks if a given peak height fidelity is required, i.e., if the permissible peak height after smoothing is 90, 95, 98, and 99% of the original peak height. We find that equations (19) and (20) are also appropriate for Lorentzians, with the coefficients given in table S4. As a rule of thumb, the kernel halfwidth m must be about 15–20% lower than for Gaussian peaks with the same FWHM.

For smoothing near boundaries, there is not much difference between Gaussian and Lorentzian peaks. Figure S9 shows the end points of the smoothed curves for different distances between the peak and the left boundary of the input data. Apart from the different peak shape, the plot is the same as Fig. 3c of the main paper and Fig. S5, and also the artifacts are similar. Smoothing with the MS and MS1 kernels (with linear extrapolation at the boundaries) results in much smaller artifacts than the other kernels (except for $n = p = 2$, where WH and MS are similar).

Table S4: Parameters for (19) and (20), for calculating filter parameters that result in a decrease of the peak height of a Lorentzian peak to 90, 95, 98, and 99% of its original value.

method	fidelity (%)	a	b	c
SGW	90	0.7469	0.2159	0.1592
	95	0.5128	0.1658	0.1397
	98	0.3274	0.1270	0.1242
	99	0.2349	0.1082	0.1157
MS	90	1.0782	0.3431	0.0703
	95	0.7503	0.2629	0.0791
	98	0.4906	0.2019	0.0821
	99	0.3608	0.1733	0.0782
MS1	90	0.3113	0.3387	0.1290
	95	0.1277	0.2557	0.1582
	98	−0.0261	0.1909	0.1873
	99	−0.1072	0.1590	0.2044
WH	90	1.5157	−0.0103	0.0125
	95	1.0618	−0.0150	0.0806
	98	0.7147	−0.0177	0.1269
	99	0.5464	−0.0186	0.1467

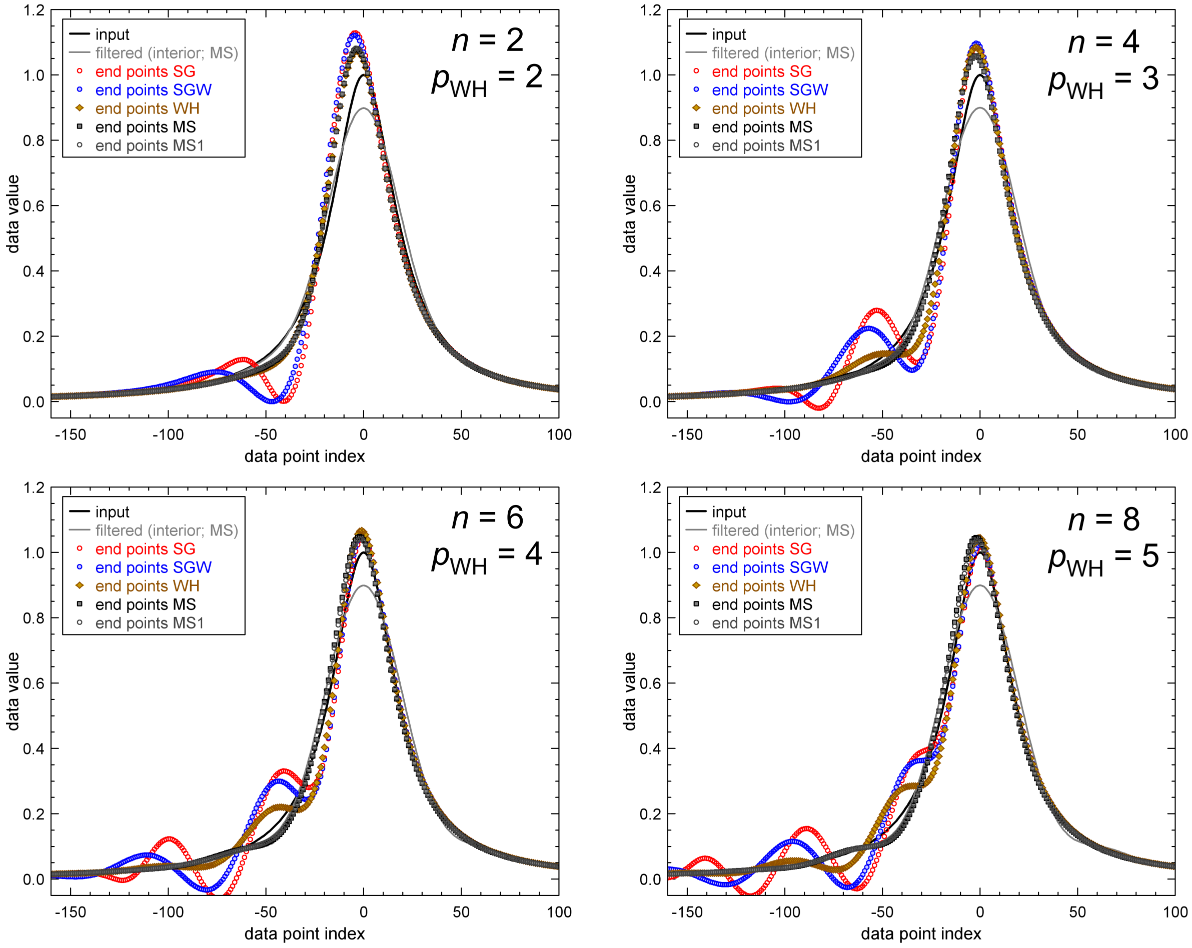

Figure S9: **End points when smoothing a near-boundary Lorentzian peak.** The figures show the filtered data points at the boundary for different positions of the boundary of the input data, as in Fig. 3c of the main paper and Fig. S5, but for a Lorentzian peak with FWHM = 40. The smoothing parameters of all filters have been chosen for a peak height fidelity of 90% when smoothing an interior peak (gray curve).
